# Supplementary material for: Unleashing the Potential of EIL Transcription Factors in Enhancing Sweet Orange Resistance to Bacterial Pathologies: Genome-Wide Identification and Expression Profiling
Source: Int J Mol Sci. 2023 Aug 10;24(16):12644. doi: 10.3390/ijms241612644 (PMC10454048; doi:10.3390/ijms241612644)
Supplement: Supplementary file 1 [file ijms-24-12644-s001.zip › ijms-2524472-supplementary.pdf]

**Supplementary Table S1.** Bioinformatics analysis of CsEIL family.

| Gene name | Gene ID | Theoretical *p*I | Mw(kDa) | Legth(aa) | localization | Gro2up | Chromosome | Exon | ORF (bp) |
|---|---|---|---|---|---|---|---|---|---|
| CsEIL 1 | orange1.1g007017m | 5.27 | 70.36 | 622 | Nuclear | EIN 3 | Chr2 : 954558-957573 | 4 | 1866 |
| CsEIL 2 | orange1.1g007173m | 5.66 | 69.57 | 615 | Nuclear | EIN | 3Chr3 : 266820-271020 | 4 | 1825 |
| CsEIL 3 | orange1.1g007662m | 5.43 | 67.09 | 595 | Nuclear | EIN 3 | chrUn : 874057-876876 | 4 | 1785 |
| CsEIL 4 | orange1.1g007983m | 5.26 | 66.22 | 583 | Nuclear | EIN 3 | Chr2 : 954558-958280 | 5 | 1749 |
| CsEIL 5 | orange1.1g009140m | 6.16 | 61.85 | 543 | Nuclear | EIN 3 | Chr3 : 259802-262917 | 4 | 1629 |
| CsEIL 6 | orange1.1g012304m | 6.11 | 52.73 | 467 | Nuclear | EIN 3 | Chr3 : 267560-271020 | 3 | 1401 |
| CsEIL 7 | orange1.1g012340m | 6.63 | 52.78 | 466 | Nuclear | EIN 3 | Chr2: 954558-957740 | 4 | 1398 |
| CsEIL 8 | orange1.1g016498m | 9.05 | 44.35 | 389 | Nuclear | EIN 3 | Chr3 : 259679-262917 | 5 | 1167 |
| CsEIL 9 | orange1.1g016840m | 5.37 | 43.96 | 382 | Nuclear | EIN 3 | Chr3: 259679-262917 | 3 | 1146 |
| CsEIL 10 | orange1.1g038853m | 5.92 | 47.35 | 323 | Nuclear | EIN | 3Chr1: 416335-417684 | 2 | 1269 |

**Supplementary Table S2.** *EIL* genes in sweet orange, Arabidopsis thaliana, rice, apple and poplar

| Gene id | Gene Name | Gene id | Gene Name |
|---|---|---|---|
| orange1.1g007017m | *CsEIL 1* | AT5G21120.1 | *AtEIL5* |
| orange1.1g007173m | *CsEIL 2* | AT5G65090.1 | *AtEIL6* |
| orange1.1g007662m | *CsEIL 3* | AT5G65100.1 | *AtEIL7* |
| orange1.1g007983m | *CsEIL 4* | LOC_Os02g36510.1 | *OsEIL1* |
| orange1.1g009140m | *CsEIL 5* | LOC_Os03g20780.1 | *OsEIL2* |
| orange1.1g012304m | *CsEIL 6* | LOC_Os03g20790.1 | *OsEIL3* |
| orange1.1g012340m | *CsEIL 7* | LOC_Os04g38400.1 | *OsEIL4* |
| orange1.1g016498m | *CsEIL 8* | LOC_Os07g12210.1 | *OsEIL5* |
| orange1.1g016840m | *CsEIL 9* | LOC_Os07g17160.1 | *OsEIL6* |
| orange1.1g038853m | *CsEIL 10* | LOC_Os07g48630.1 | *OsEIL7* |
| AT1G73730.1 | *AtEIL1* | LOC_Os07g48630.2 | *OsEIL8* |
| AT2G27050.1 | *AtEIL2* | LOC_Os08g39830.1 | *OsEIL9* |
| AT3G20770.1 | *AtEIL3* | LOC_Os08g39830.2 | *OsEIL10* |
| AT5G10120.1 | *AtEIL4* | LOC_Os09g31400.1 | *OsEIL11* |
| NM_001328892.2 | *MdEIL1* | XM_024610055.4 | *PtEIL4* |
| OU696679.1 | *MdEIL2* | XM_002299212.3 | *PtEIL5* |
| XM_008347911.3 | *MdEIL3* | XM_002307066.3 | *PtEIL6* |
| XM_008356616.3 | *MdEIL4* | XM_002307066.3 | *PtEIL7* |
| XM_017335709.1 | *MdEIL5* | XM_002312805.3 | *PtEIL8* |
| XM_017335709.2 | *MdEIL6* | XM_002312805.3 | *PtEIL9* |
| XM_029093268.1 | *MdEIL7* | XM_024598281.1 | *PtEIL10* |
| OU696691.1 | *MdEIL8* | XM_024598281.1 | *PtEIL11* |
| OU744993.1 | *MdEIL9* | XM_024598281.1 | *PtEIL12* |
| OU744998.1 | *MdEIL10* | XM_024600202.1 | *PtEIL13* |
| OU745005.1 | *MdEIL11* | XM_024605398.1 | *PtEIL14* |
| XM_008347911.3 | *MdEIL12* | XM_024605398.1 | *PtEIL15* |
| XM_008347917.3 | *MdEIL13* | XM_024606204.1 | *PtEIL16* |
| XM_008363268.3 | *MdEIL14* | XM_024606204.1 | *PtEIL17* |
| XM_008366362.3 | *MdEIL15* | XM_024606204.1 | *PtEIL18* |
| XM_008367942.3 | *MdEIL16* | XM_024606205.1 | *PtEIL19* |
| XM_029093454.1 | *MdEIL17* | XM_024606205.1 | *PtEIL20* |
| XM_029105228.1 | *MdEIL18* | XM_024606205.1 | *PtEIL21* |
| XM_029105228.1 | *MdEIL19* | XM_024606205.2 | *PtEIL22* |
| XM_029105229.1 | *MdEIL20* | XM_024606205.3 | *PtEIL23* |
| XM_029105230.1 | *MdEIL21* | XM_024607301.1 | *PtEIL24* |

| Gene id | Gene Name | Gene id | Gene Name |
|---|---|---|---|
| XM_029106284.1 | *MdEIL22* | XM_024607301.2 | *PtEIL25* |
| XM_024610055.1 | *PtEIL1* | XM_024608163.1 | *PtEIL26* |
| XM_024610055.2 | *PtEIL2* | XM_024608164.1 | *PtEIL27* |
| XM_024610055.3 | *PtEIL3* | XM_024610055.1 | *PtEIL28* |

**Supplementary Table S3.** Collinearity analysis of sweet orange, Arabidopsis thaliana, rice, apple and poplar

| gene id | gene name | gene id | gene name | chr id | Cs start/end | At start/end |
|---|---|---|---|---|---|---|
| orange1.1g007017m | CsEIL1 | AT2G27050.1 | AtEIL2 | Cs chr2/At chr2 | 954558-957573 | 11545656-11548293 |
| orange1.1g007983m | CsEIL4 | AT2G27050.1 | AtEIL2 | Cs chr2/At chr2 | 954558-958280 | 11545656-11548293 |
| orange1.1g012340m | CsEIL7 | AT2G27050.1 | AtEIL2 | Cs chr2/At chr2 | 954558-957740 | 11545656-11548293 |
| orange1.1g038853m | CsEIL10 | AT5G65090.1 | AtEIL6 | Cs chr1/At chr5 | 416335-417684 | 7182589-7184342 |
| orange1.1g007017m | CsEIL1 | AT5G21120.1 | AtEIL4 | Cs chr2/At chr5 | 954558-957573 | 26003882-26006851 |
| orange1.1g007983m | CsEIL4 | AT5G21120.1 | AtEIL4 | Cs chr2/At chr5 | 954558-958280 | 26003882-26006851 |
| orange1.1g012340m | CsEIL7 | AT5G21120.1 | AtEIL4 | Cs chr2/At chr5 | 954558-957740 | 26003882-26006851 |
| orange1.1g007173m | CsEIL2 | XM_008347917.3 | MdEIL12 | Cs chr3/Md chr2 | 266820-271020 | 32069916-32073820 |
| orange1.1g009140m | CsEIL5 | XM_008347917.3 | MdEIL12 | Cs chr3/Md chr2 | 259802-262917 | 32069916-32073820 |
| orange1.1g012304m | CsEIL6 | XM_008347917.3 | MdEIL12 | Cs chr3/Md chr2 | 267560-271020 | 32069916-32073820 |
| orange1.1g016498m | CsEIL8 | XM_008347917.3 | MdEIL12 | Cs chr3/Md chr2 | 259679-262917 | 32069916-32073820 |
| orange1.1g016840m | CsEIL9 | XM_008347917.3 | MdEIL12 | Cs chr3/Md chr2 | 259679-262917 | 32069916-32073820 |
| orange1.1g007173m | CsEIL2 | XM_029105228.1 | MdEIL19 | Cs chr3/Md chr7 | 266820-271020 | 4954483-4958869 |
| orange1.1g009140m | CsEIL5 | XM_029105228.1 | MdEIL19 | Cs chr3/Md chr7 | 259802-262917 | 4954483-4958869 |
| orange1.1g012304m | CsEIL6 | XM_029105228.1 | MdEIL19 | Cs chr3/Md chr7 | 267560-271020 | 4954483-4958869 |
| orange1.1g016498m | CsEIL8 | XM_029105228.1 | MdEIL19 | Cs chr3/Md chr7 | 259679-262917 | 4954483-4958869 |
| orange1.1g016840m | CsEIL9 | XM_029105228.1 | MdEIL19 | Cs chr3/Md chr7 | 259679-262917 | 4954483-4958869 |
| orange1.1g038853m | CsEIL10 | XM_008366362.3 | MdEIL15 | Cs chr1/Md chr15 | 416335-417684 | 35505799-35513442 |
| orange1.1g007017m | CsEIL1 | XM_024605398.1 | PtEIL7 | Cs chr2/Pt chr9 | 954558-957573 | 11613079-11617694 |
| orange1.1g007983m | CsEIL4 | XM_024605398.1 | PtEIL7 | Cs chr2/Pt chr9 | 954558-958280 | 11613079-11617694 |

| gene id | gene name | gene id | gene name | chr id | Cs start/end | At start/end |
|---|---|---|---|---|---|---|
| orange1.1g012340m | CsEIL7 | XM_024605398.1 | PtEIL7 | Cs chr2/Pt chr9 | 954558-957740 | 11613079-11617694 |
| orange1.1g007173m | CsEIL2 | XM_024606204.1 | PtEIL12 | Cs chr3/Pt chr8 | 266820-271020 | 626979-631297 |
| orange1.1g009140m | CsEIL5 | XM_024606204.1 | PtEIL12 | Cs chr3/Pt chr8 | 259802-262917 | 626979-631297 |
| orange1.1g012304m | CsEIL6 | XM_024606204.1 | PtEIL12 | Cs chr3/Pt chr8 | 267560-271020 | 626979-631297 |
| orange1.1g016498m | CsEIL8 | XM_024606204.1 | PtEIL12 | Cs chr3/Pt chr8 | 259679-262917 | 626979-631297 |
| orange1.1g016840m | CsEIL9 | XM_024606204.1 | PtEIL12 | Cs chr3/Pt chr8 | 259679-262917 | 626979-631297 |
| orange1.1g007173m | CsEIL2 | XM_024610055.1 | PtEIL24 | Cs chr3/Pt chr10 | 266820-271020 | 4954483-4958869 |
| orange1.1g009140m | CsEIL5 | XM_024610055.1 | PtEIL24 | Cs chr3/Pt chr10 | 259802-262917 | 4954483-4958869 |
| orange1.1g012304m | CsEIL6 | XM_024610055.1 | PtEIL24 | Cs chr3/Pt chr10 | 267560-271020 | 35505799-35513442 |
| orange1.1g016498m | CsEIL8 | XM_024610055.1 | PtEIL24 | Cs chr3/Pt chr10 | 259679-262917 | 22117946-22122347 |
| orange1.1g016840m | CsEIL9 | XM_024610055.1 | PtEIL24 | Cs chr3/Pt chr10 | 259679-262917 | 22117946-22122347 |
| orange1.1g038853m | CsEIL10 | XM_002307066.3 | PtEIL6 | Cs chr1/Pt chr5 | 416335-417684 | 5747051-5751372 |
| orange1.1g038853m | CsEIL10 | XM_024605398.1 | PtEIL11 | Cs chr1/Pt chr7 | 416335-417684 | 11613079-11617694 |
| orange1.1g007662m | CsEIL3 | XM_024598281.1 | PtEIL8 | Cs chrUn/Pt chr3 | 874057-876876 | 20939771-20942987 |

**Supplementary Table S4.** List of qRT-PCR primers

| Gene ID | Forward primer (5'-3') | Reverse primer (5'-3') |
|---|---|---|
| *CsEIL1* | ACTCTTGTCGGCTCTCATGC | TGATCACTGCCGTCAACACA |
| *CsEIL2* | ATTCGACCGTAATGGCCCTG | GCTTGGAAAGTGGGGGTTCT |
| *CsEIL3* | CCAGCAGCCATAGCGAAGTA | TTGGAGAAAGGAGTTCCGCC |
| *CsEIL4* | GTCCACCCCCGTACAAGAAG | CGGGAGTGGCTCATTCATCA |
| *CsEIL5* | GCTTGGAAAGTGGGGGTTCT | CCGAAAGCTTGTTCGCCAAT |
| *CsEIL6* | ACAATCTCCGTGAGTGGTGG | AGAGTTGCAAGACACCACCC |
| *CsEIL7* | CCGCTGGAAAAAGGTGTTGC | TGGCGGTAATTAACCA |
| *CsEIL8* | TGCTAAGCAGCGTCAATCCC | CCTGTGAGTTTCCCCAGTGT |
| *CsEIL9* | GAAGGAGAGGCGTTTCTGGA | ATGTCTCGGGCACAAGATGG |
| *CsEIL10* | ACTCTTGTCGGCTCTCATGC | CAATCAGGAGGAGGCCTTGG |
| *CsCOX* | GTATGCCACGTCGCATTCCAGA | GCCAAAACTGCTAAGGGCATTC |

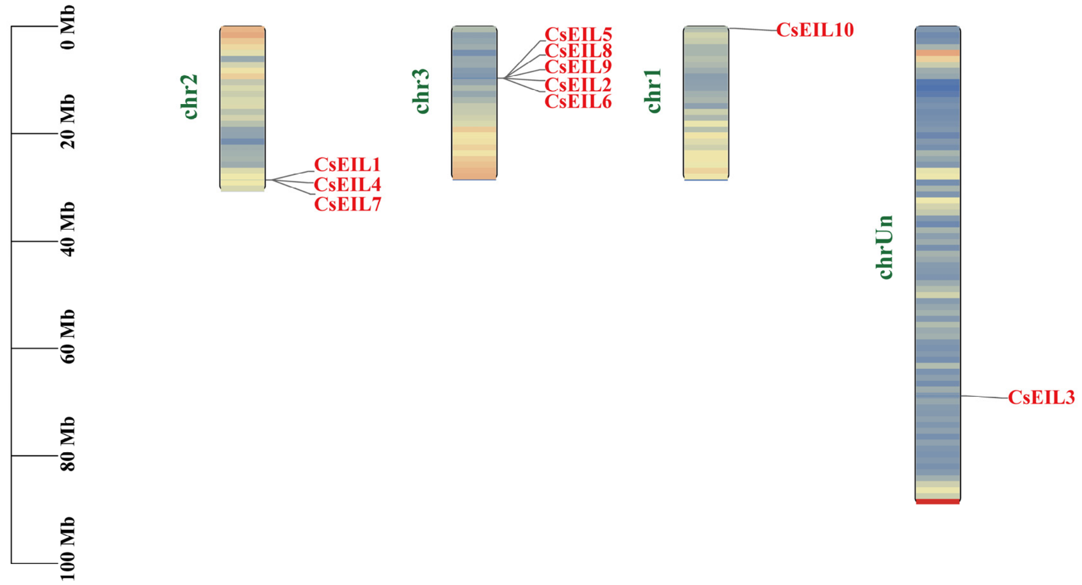


**Supplementary Figure S1.** Chromosome localization of the *CsEIL* genes.

The size of chromosomes is estimated based on their relative lengths, and chromosome numbers are shown on the left of each chromosome. Gene density is represented by a color bar (a blue to red scale indicates low to high gene density).

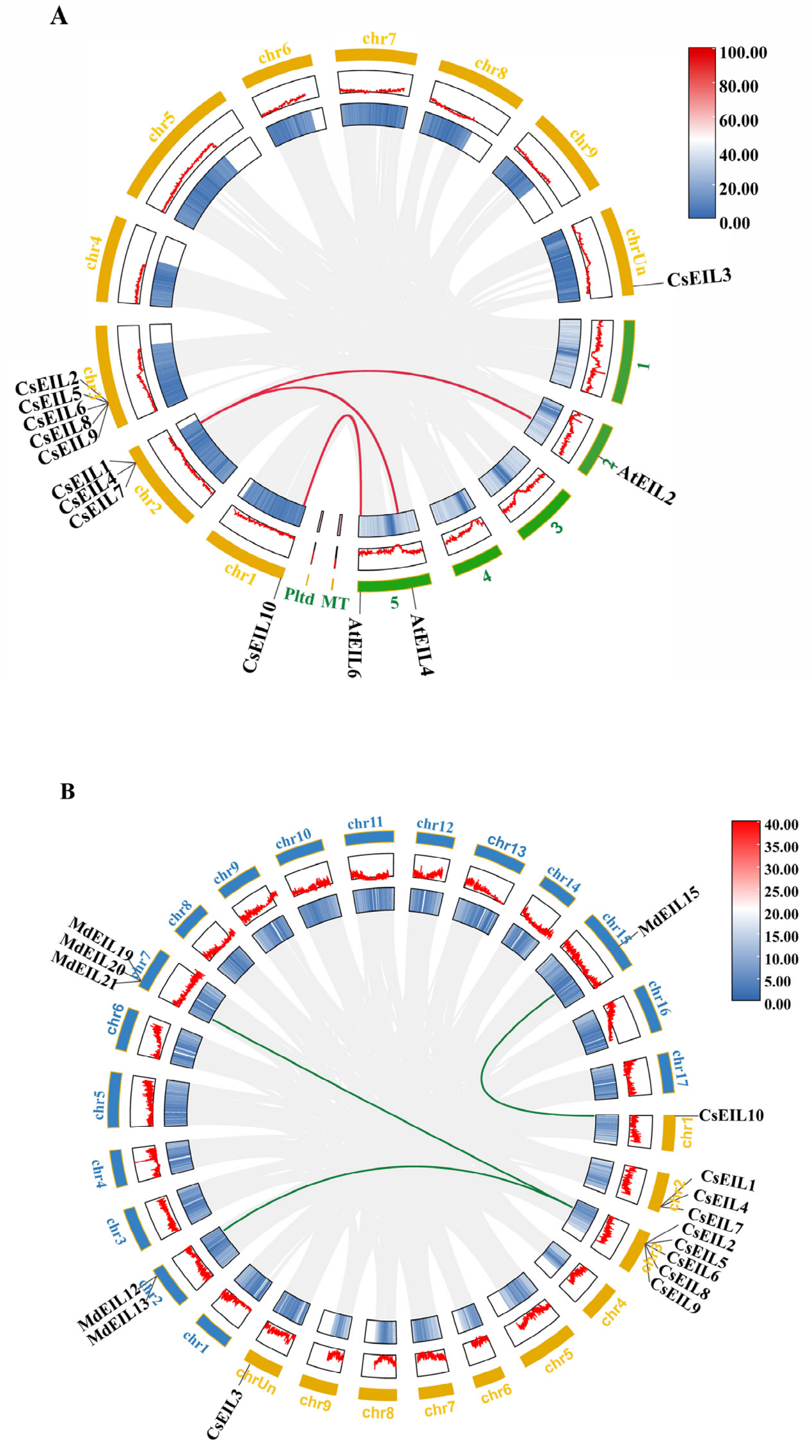


**Supplementary Figure S2.** Replication and collinearity analysis of *EIL* genes in sweet orange, Arabidopsis, and apple.

Supplementary Figure S2A shows the collinear relationship between sweet orange and Arabidopsis, while Supplementary Figure S2B shows the collinear relationship between sweet orange and apple. The gray lines represent all collinear regions, and the other colored lines represent homologous gene pairs (the blue to red scale indicates the gene density from low to high)

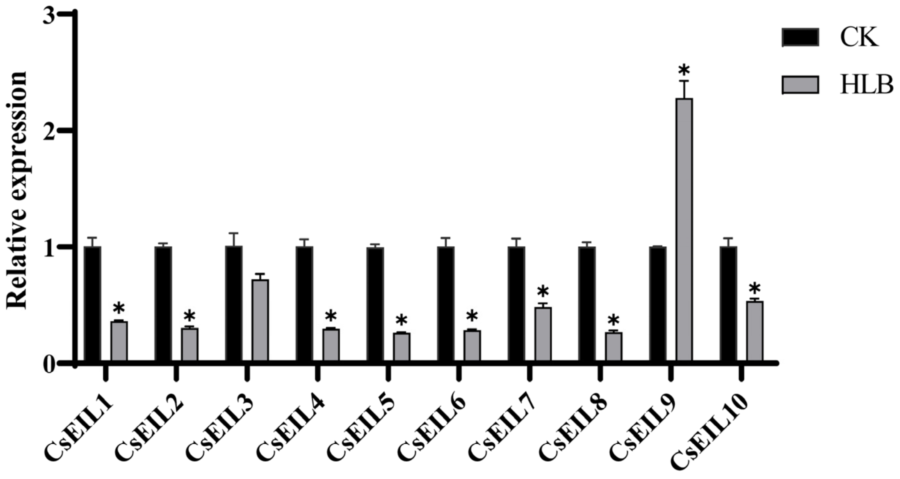


**Supplementary Figure S3.** Expression level of *CsEIL* genes after *Candidatus* Liberibacter asiaticus inoculation.

CK is a healthy plant and HLB is a *C*Las-infected plant. The experiment was conducted with three biological replicates in all panels, and similar outcomes were observed. The asterisk (*) indicates a significant difference ($P$<0.05).

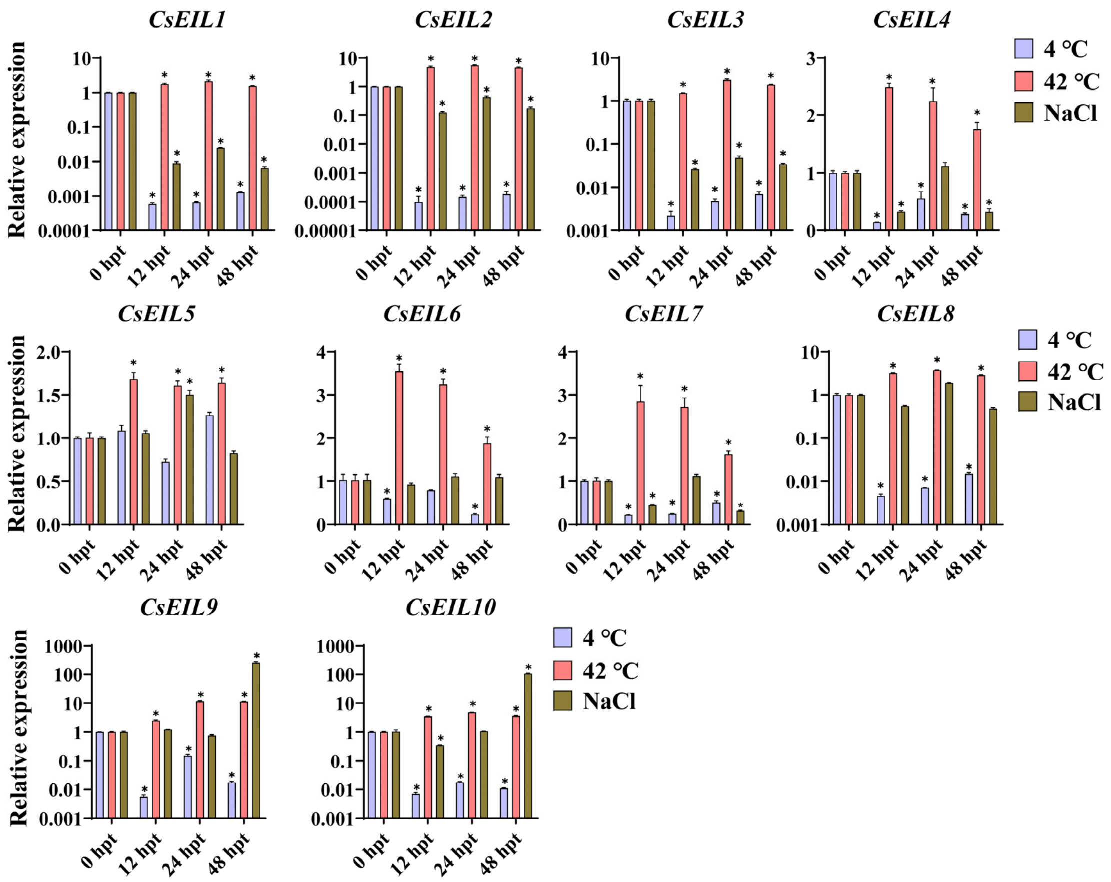


**Supplementary Figure S4.** Expression levels of *CsEIL* genes under different abiotic stresses.

The X-axis represents the different time points after exposure to various abiotic stress treatments, while the Y-axis shows the relative gene expression levels of *CsEIL*. The purple bar represents treatment at 4°C, the pink bar represents treatment at 42°C, and the brown bar represents NaCl treatment. The experiment was performed with three biological replicates, and the asterisk (*) indicates significant difference ($P<0.05$).
